# A global map of hemispheric influenza vaccine recommendations based on local patterns of viral circulation

**DOI:** 10.1038/srep17214

**Published:** 2015-12-01

**Authors:** Wladimir J. Alonso, Christine Yu, Cecile Viboud, Stephanie A. Richard, Cynthia Schuck-Paim, Lone Simonsen, Wyller A. Mello, Mark A. Miller

**Affiliations:** 1 National Institutes of Health, Fogarty International Center, Bethesda, MD, 20892, USA; 2 Wolfson College, Linton Road, OX26UD, Oxford, UK; 3 George Washington University, Milken Institute School of Public Health, Washington, DC 20052, USA; 4 Evandro Chagas Institute, WHO Global Influenza Surveillance Network, Para, Brazil

## Abstract

Both the Northern and the Southern Hemisphere annual WHO influenza vaccine recommendations are designed to ensure vaccine delivery before the winter-time peak of viral circulation in each hemisphere. However, influenza seasonal patterns are highly diverse in tropical countries and may be out of phase with the WHO recommendations for their respective hemisphere. We modelled the peak timing of influenza activity for 125 countries using laboratory-based surveillance data from the WHO’s FLUNET database and compared it with the influenza hemispheric recommendations in place. Influenza vaccine recommendations for respectively 25% and 39% of the Northern and Southern Hemisphere countries were out of phase with peak influenza circulation in their corresponding hemisphere (62% and 53%, respectively, when the analysis was limited to the 52 countries in the tropical belt). These results indicate that routine influenza immunization efforts should be closely tailored to local patterns of viral circulation, rather than a country’s hemispheric position.

Influenza is a major respiratory pathogen with a recognized global disease burden in epidemic and pandemic seasons^1^. A constant race between the host immune system and influenza virus evolution requires annual revaccination of populations, in particular the elderly, pregnant women, children, and those with chronic conditions, with periodically updated tri- or quadri-valent influenza vaccines that aim to provide protection against 3 dominant types/subtypes (A/H3, A/H1, B)^2^. Routine vaccinations efforts have been in place for several decades in developed countries to immunize populations before the winter influenza season, while vaccination has recently gained traction in low and middle income countries^3^.

The World Health Organization (WHO) has established the Global Influenza Surveillance and Response System (GISRS) to inform the WHO influenza vaccine committee, who have met twice a year since 1999 to decide on the antigenic composition of influenza vaccines for the next influenza season in each hemisphere^6^,^7^. To establish the Northern Hemisphere (NH) recommendations, globally circulating influenza virus strains are reviewed every February, so that the vaccine can be distributed between September and October, in advance of the winter influenza season. Likewise, for the Southern Hemisphere (SH) recommendations, influenza virus circulation patterns are reviewed each September, so that vaccination usually takes place between March and April of the following year. The current six-month delay between the WHO expert recommendations and vaccine availability is due to limitations of the current technology used in the manufacturing process, which represents a severe challenge for vaccine efficacy. On occasion, the antigen(s) present in the vaccine are mismatched with the circulating strains, causing important vaccine failures^8^, as documented in the recent 2014-15 NH season^9^,^10^.

Given the current limitations in vaccine production, the goal is to optimize vaccine formulation and timing of administration in each geographic area. This is particularly challenging for tropical regions, where influenza activity are frequently out of phase with the dynamics predicted for their hemispheric group^11^,^12^,^13^,^14^,^15^,^16^,^17^. In fact, recent studies have shown that the optimal timing for routine influenza vaccination recommendations does not correspond to the one expected for their hemisphere in tropical regions of South and Central America^11^,^12^,^17^, Southern and South-Eastern Asia^14^, China^15^, and Africa^18^,^19^. These studies have been important to guide local routine influenza vaccination programs; however these were limited to a handful of countries. Here we extend this approach by mapping influenza seasonality in 125 countries and synthesize available epidemiological evidence to make global vaccine recommendations tailored to the local epidemiology and geography of the disease.

## Results

**Figure 1:**
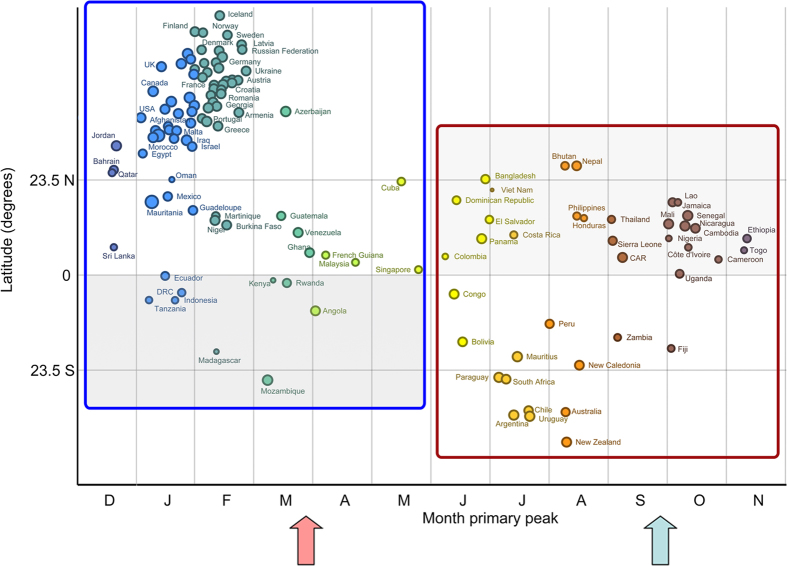
Timing of the primary peak of influenza detection (2010–2014), by country, against the latitudinal position of the capital city. The size of points corresponds to the amplitude of influenza seasonality. Colors are used to highlight differences in peak timing. Arrows indicate the typical timing of delivery of the Southern Hemisphere (red) and Northern Hemisphere (blue) vaccines. Countries in the blue and red boxes should adopt the Northern and Southern Hemispheric vaccines, respectively. In each box the area with the darker background highlights countries that should opt for the vaccine recommended for the opposite hemisphere.

The timing of primary peaks obtained from the 2010–2014 influenza virus circulation time series of each country relative to their capital cities’ latitude is shown in Fig. 1. Overall, peak influenza circulation patterns in temperate countries were well aligned with the winter season - mostly between January and February in the Northern Hemisphere (blue and green circles above the northern tropical line in the figure), and July and August in the Southern Hemisphere (bright and dark orange circles below the Southern tropical line in the figure). Conversely, the timing of peak influenza activity in tropical countries had little regard for hemispheric position, and peak influenza activity was distributed unpredictably throughout the year.

**Figure 2:**
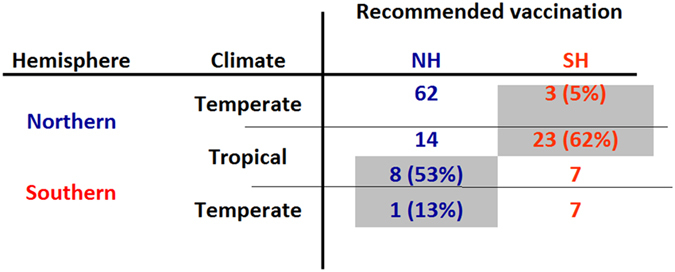
Optimal influenza vaccine recommendations for 125 countries contributing surveillance data (NH: Northern Hemisphere; SH: Southern Hemisphere).

Based on the estimated timing of the peak influenza activity, we classified all countries with respect to the vaccine they should adopt (Figs. 1, 2, S1 Table). In total, 26 countries of the Northern Hemisphere should opt for the vaccination recommendation currently targeted at Southern Hemisphere countries (the countries in the shaded area within the red box in Fig. 1 and in Fig. 1: Bhutan, Nepal, Bangladesh, Viet Nam, Dominican Republic, Jamaica, Lao People’s Democratic Republic, Senegal, Philippines, Honduras, Thailand, El Salvador, Mali, Nicaragua, Cambodia, Costa Rica, Nigeria, Ethiopia, Panama, Sierra Leone, Côte d’Ivoire, Togo, Colombia, Central African Republic, Cameroon, Uganda). l. On the other hand, nine countries of Southern Hemisphere (Ecuador, Kenya, Rwanda, Democratic Republic of the Congo, United Republic of Tanzania, Indonesia, Angola, Madagascar, Mozambique) should opt for the vaccine targeted for the Northern Hemisphere (shaded area within the blue box in Fig. 2, Fig.1 and S1 Table). These ‘out-of-phase’ recommendations represent 62% and 53% of countries in the tropical belt of the Northern and Southern Hemisphere. In other words, most countries in this region should receive the vaccine initially recommended for the opposite hemisphere. A few countries with their capital located outside tropical belt (Bhutan, Nepal and Bangladesh in the Northern Hemisphere and Mozambique in the Southern Hemisphere) should also opt for the vaccine for the opposite hemisphere.

## Discussion

We have presented here the first global map of hemispheric influenza recommendations based on analysis of laboratory-confirmed epidemiological data for 125 countries. Our results show that, unexpectedly, more than half of the countries in the tropical belt would achieve better vaccine coverage relative to their epidemic patterns by adopting the vaccine formulation developed for the opposite hemisphere. Our data shows that the criteria for determining the most appropriate vaccine recommendation should not depend on the hemispheric location of a country, but instead on local epidemiological information. Such data could be from national influenza surveillance systems or, if such data are not available, from neighboring populations. These findings are of critical importance to countries in these regions that are currently providing, or envisioning, annual influenza vaccine, as failure to adapt vaccine calendars tailored to local circulation patterns is likely to severely impair the success of immunization efforts.

Our study was very inclusive in that we used information from a large and diverse set of countries; however we were limited by the lack of availability of subnational data. Studies in Brazil and China showed the importance of regional heterogeneity in influenza timing in large countries spanning temperate and tropical regions, indicating the need for staggered timing of vaccination^11^,^12^,^15^. Subsequent studies confirmed that these findings were valid for other tropical countries^20^. Detailed antigenic characterization should also be performed whenever possible in order to carefully study the temporal patterns of emergence of novel drift variants in each hemisphere and each country and the match with vaccine strains (e.g.^8^,^21^). It is important to note that seasonality is not well defined (or inexistent) in many tropical regions^1^,^19^. Therefore, although our results show the best possible vaccination options based on the data currently available, confidence intervals for the peak timing in those regions would be very broad (despite previous attempts^11^, the methodology for obtaining confidence intervals in this context is not well established yet). We encourage our readers to inspect and reproduce our analyses using different assumptions and approaches (e.g. using raw data instead of polynomial-detrended data, or consider different time-windows). To this end we have made the underlying epidemiological time series data available at www.epipoi.info/flunet. The free software used for analyses can be downloaded at www.epipoi.info. We also encourage countries with regions in tropical areas to investigate sub-national patterns of influenza and other respiratory virus circulation, to estimate the burden of those pathogens, and how they relate to diverse climatology and connectivity patterns.

Future work to obtain the optimal influenza vaccine match in any country should combine epidemiological and antigenic (and/or phylogenetic) information to evaluate the pathways of virus migration and evolution between regions, countries, and districts. One excellent way to make such data available for analysis would be for WHOs FLUNET database to include information on dominant drift variants were dominant on a weekly or seasonal basis.

Also, further studies should elucidate the social, geographic and demographic factors underlying aberrations in seasonality in some countries^19^. The study of population and environmental drivers of influenza circulation should also improve the spatial and temporal resolution of recommendations. Finally, although time series analysis of long-term influenza records can be insightful to hypothesize possible routes of influenza diffusion between states, countries and regions (eg,^19^,^24^), precise migration pathways can only be inferred from phylogeographic analyses of influenza viral sequences^7^,^23^.

By investigating influenza seasonality for most of the world (including countries that have not been the focus of such analyses before) and making the data and analytical tools used available, we believe this study should contribute to the design of influenza vaccination policies, as well as to further research at national and regional scales.

## Methods

### Epidemiological Data

**Figure 3:**
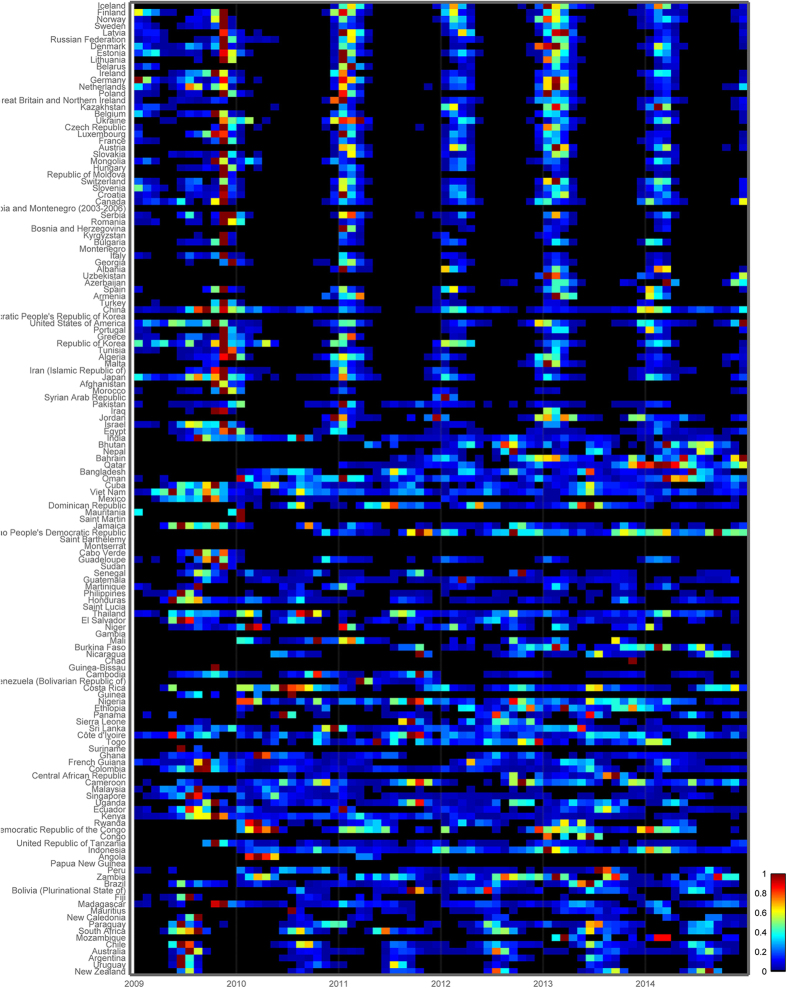
Heat map of monthly influenza virus incidence patterns in 125 countries, 2009-2014, sorted by latitude of the capital cities. Color bar represents the intensity of influenza incidence, from high (red) to low (blue). Monthly incidence counts were standardized annually, and shown as the proportion of the maximum number of cases in a month for that country and period (hence, months with the maximum number of cases for a given year were assigned the value 1). Year 2009 was excluded from seasonality analyses due to the A/H1N1 pandemic emergence. Data source: FluNet^5^,^26^. Visualization: Epipoi^28^.

We compiled laboratory-confirmed influenza cases by virus type (seasonal H1N1, H1N1pdm09, H3N2, B and unsubtyped) for 144 countries from the FluNet database, the global influenza surveillance system maintained by WHO^25^,^26^ (http://www.who.int/influenza/gisrs_laboratory/flunet/en). There was a surge of virus-surveillance after the 2009 influenza pandemic; therefore, we focused on the post-pandemic period 2010–2014 (Fig. 3), when reporting was stable and stronger (2009 was excluded because it was markedly atypical, and 2015 because it is incomplete at the time of this writing). Information was not available at the sub-national level, therefore we generated monthly influenza time-series for each country, with at least 20 virus specimens reported for this period. We also excluded Brazil, India and China as they are large countries spanning temperate and tropical regions, where subnational studies were previously carried out^11^,^12^,^15^. A total of 125 countries qualified for these analyses.

### Statistical Analyses

For each country, we performed time-series analyses to infer the month of peak timing of influenza circulation. For each time-series, we first obtained the periodic annual function (PAF) of the epidemiological time series after de-trending with a quadratic polynomial, and summing up the annual, semi-annual and quarterly harmonics as obtained by Fourier decomposition^11^,^27^,^28^. The timing of the primary (major) peak of the PAF was obtained, and plotted against the latitude of each country’s capital (S1 Table), so as to compare the timing of peak influenza activity against that of the two hemispheric vaccine recommendations. Then, we determined whether each country should adopt the NH or SH hemisphere vaccine recommendation. We reasoned that the optimal strategy for any country was to use the most recently available vaccine formulation with respect to local peak influenza timing, as long as the vaccine was available at least two months prior to the peak (early April for the SH formulation, early October for the NH formulation). We estimated two months as the minimum amount of time required to ensure proper vaccine delivery and immunization, and develop immunity. Analyses and figures were generated using the freely available analytical software Epipoi^28^.

## Additional Information

**How to cite this article**: Alonso, W. J. *et al.* A global map of hemispheric influenza vaccine recommendations based on local patterns of viral circulation. *Sci. Rep.* **5**, 17214; doi: 10.1038/srep17214 (2015).

## Supplementary Material

Supplementary Information
