# Supplementary material for: A global map of hemispheric influenza vaccine recommendations based on local patterns of viral circulation
Source: Sci Rep. 2015 Dec 1;5:17214. doi: 10.1038/srep17214 (PMC4664865; doi:10.1038/srep17214)
Supplement: Supplementary Information [file srep17214-s1.pdf]

supplementary information to the article

A global map of hemispheric influenza vaccine recommendations based on local patterns of viral circulation

Wladimir J. Alonso[1*], Christine Yu[3], Cecile Viboud[1], Stephanie A. Richard[1], Cynthia Schuck-Paim[2], Lone Simonsen[3], Wyller A. Mello[4], & Mark A. Miller[1]

[*]wladimir.alonso@nih.gov

[1] Fogarty International Center, National Institutes of Health, MD, USA
[2] Wolfson College, Oxford, UK
[3] George Washington University, DC, USA
[4] Evandro Chagas Institute, WHO Global Influenza Surveillance Network, Para, Brazil

2015

Table S1. Countries included in the analysis, month of primary peak and most appropriate vaccine (most recent vaccine at least two months before the primary peak) . Countries that should adopt the vaccine recommended for the opposite hemisphere (as measured by their capital latitude) are highlighted with a grey background. Key of colors follow the ones used in the article

Country	Latitude Capital	Classification *	Hemisphere	Month primary peak**	Correct vaccine
Iceland	*64.1*	*Temperate*	Northern	2.4	**NHR**
Finland	*60.2*	*Temperate*	Northern	2.0	**NHR**
Norway	*59.9*	*Temperate*	Northern	2.1	**NHR**
Sweden	*59.3*	*Temperate*	Northern	2.6	**NHR**
Latvia	*56.9*	*Temperate*	Northern	2.8	**NHR**
Russian Federation	*55.7*	*Temperate*	Northern	2.8	**NHR**
Denmark	*55.7*	*Temperate*	Northern	2.1	**NHR**
Estonia	*55.4*	*Temperate*	Northern	2.4	**NHR**
Lithuania	*54.7*	*Temperate*	Northern	1.9	**NHR**
Belarus	*53.9*	*Temperate*	Northern	2.5	**NHR**
Ireland	*53.3*	*Temperate*	Northern	1.9	**NHR**
Germany	*52.5*	*Temperate*	Northern	2.4	**NHR**
Netherlands	*52.4*	*Temperate*	Northern	2.2	**NHR**
Poland	*52.2*	*Temperate*	Northern	1.8	**NHR**
UK	*51.5*	*Temperate*	Northern	1.4	**NHR**
Kazakhstan	*51.2*	*Temperate*	Northern	2.4	**NHR**
Belgium	*50.9*	*Temperate*	Northern	2.0	**NHR**
Ukraine	*50.5*	*Temperate*	Northern	2.9	**NHR**
Czech Republic	*50.1*	*Temperate*	Northern	2.2	**NHR**
Luxembourg	*49.6*	*Temperate*	Northern	2.0	**NHR**
France	*48.9*	*Temperate*	Northern	2.1	**NHR**
Austria	*48.2*	*Temperate*	Northern	2.6	**NHR**
Slovakia	*48.1*	*Temperate*	Northern	2.7	**NHR**
Mongolia	*47.9*	*Temperate*	Northern	2.5	**NHR**
Hungary	*47.5*	*Temperate*	Northern	2.6	**NHR**
Republic of Moldova	*47.0*	*Temperate*	Northern	2.5	**NHR**
Switzerland	*46.9*	*Temperate*	Northern	2.3	**NHR**
Slovenia	*46.1*	*Temperate*	Northern	2.3	**NHR**
Croatia	*45.8*	*Temperate*	Northern	2.5	**NHR**
Canada	*45.4*	*Temperate*	Northern	1.3	**NHR**
Serbia	*44.8*	*Temperate*	Northern	2.5	**NHR**
Romania	*44.4*	*Temperate*	Northern	2.3	**NHR**
Bosnia and Herzeg.	*43.9*	*Temperate*	Northern	1.9	**NHR**

Kyrgyzstan	*42.9*	*Temperate*	Northern	1.6	**NHR**
Bulgaria	*42.7*	*Temperate*	Northern	2.3	**NHR**
Italy	*41.9*	*Temperate*	Northern	2.0	**NHR**
Georgia	*41.7*	*Temperate*	Northern	2.4	**NHR**
Albania	*41.3*	*Temperate*	Northern	2.2	**NHR**
Uzbekistan	*41.0*	*Temperate*	Northern	1.5	**NHR**
Azerbaijan	*40.4*	*Temperate*	Northern	3.5	**NHR**
Spain	*40.4*	*Temperate*	Northern	2.0	**NHR**
Armenia	*40.2*	*Temperate*	Northern	2.8	**NHR**
Turkey	*39.9*	*Temperate*	Northern	1.7	**NHR**
USA	*38.9*	*Temperate*	Northern	1.1	**NHR**
Portugal	*38.7*	*Temperate*	Northern	2.1	**NHR**
Greece	*38.0*	*Temperate*	Northern	2.2	**NHR**
Republic of Korea	*37.6*	*Temperate*	Northern	1.9	**NHR**
Tunisia	*36.8*	*Temperate*	Northern	2.4	**NHR**
Algeria	*36.8*	*Temperate*	Northern	1.6	**NHR**
Malta	*35.9*	*Temperate*	Northern	1.6	**NHR**
Iran	*35.7*	*Temperate*	Northern	1.3	**NHR**
Japan	*35.7*	*Temperate*	Northern	1.7	**NHR**
Afghanistan	*34.5*	*Temperate*	Northern	1.4	**NHR**
Morocco	*34.0*	*Temperate*	Northern	1.3	**NHR**
Pakistan	*33.7*	*Temperate*	Northern	1.7	**NHR**
Iraq	*33.3*	*Temperate*	Northern	1.9	**NHR**
Jordan	*32.0*	*Temperate*	Northern	12.7	**NHR**
Israel	*31.8*	*Temperate*	Northern	2.0	**NHR**
Egypt	*30.0*	*Temperate*	Northern	1.1	**NHR**
Bhutan	*27.0*	*Temperate*	Northern	8.3	**SHR**
Nepal	*27.0*	*Temperate*	Northern	8.5	**SHR**
Bahrain	*26.0*	*Temperate*	Northern	12.6	**NHR**
Qatar	*25.3*	*Temperate*	Northern	12.6	**NHR**
Bangladesh	*23.7*	*Temperate*	Northern	6.9	**SHR**
Oman	*23.6*	*Temperate*	Northern	1.6	**NHR**
**Cuba**	**23.1**	**Tropical**	**Northern**	5.5	**NHR**
**Viet Nam**	**21.0**	**Tropical**	**Northern**	7.0	**SHR**
**Mexico**	**19.4**	**Tropical**	**Northern**	1.5	**NHR**
**Dominican Republic**	**18.5**	**Tropical**	**Northern**	6.4	**SHR**
**Mauritania**	**18.1**	**Tropical**	**Northern**	1.3	**NHR**
**Jamaica**	**18.0**	**Tropical**	**Northern**	10.1	**SHR**
**Laos**	**18.0**	**Tropical**	**Northern**	10.2	**SHR**
**Guadeloupe**	**16.0**	**Tropical**	**Northern**	2.0	**NHR**

Senegal	14.7	Tropical	Northern	10.3	SHR
Guatemala	14.6	Tropical	Northern	3.5	NHR
Martinique	14.6	Tropical	Northern	2.4	NHR
Philippines	14.6	Tropical	Northern	8.5	SHR
Honduras	14.1	Tropical	Northern	8.6	SHR
Thailand	13.8	Tropical	Northern	9.1	SHR
El Salvador	13.7	Tropical	Northern	7.0	SHR
Niger	13.5	Tropical	Northern	2.3	NHR
Mali	12.7	Tropical	Northern	10.0	SHR
Burkina Faso	12.4	Tropical	Northern	2.5	NHR
Nicaragua	12.1	Tropical	Northern	10.3	SHR
Cambodia	11.6	Tropical	Northern	10.5	SHR
Venezuela	10.5	Tropical	Northern	3.8	NHR
Costa Rica	9.9	Tropical	Northern	7.4	SHR
Nigeria	9.1	Tropical	Northern	10.0	SHR
Ethiopia	9.0	Tropical	Northern	11.4	SHR
Panama	9.0	Tropical	Northern	6.9	SHR
Sierra Leone	8.5	Tropical	Northern	9.1	SHR
Sri Lanka	6.9	Tropical	Northern	12.6	NHR
Côte d'Ivoire	6.9	Tropical	Northern	10.4	SHR
Togo	6.1	Tropical	Northern	11.3	SHR
Ghana	5.6	Tropical	Northern	3.9	NHR
French Guiana	4.9	Tropical	Northern	4.2	NHR
Colombia	4.6	Tropical	Northern	6.2	SHR
Cent. Afr. Republic	4.4	Tropical	Northern	9.2	SHR
Cameroon	3.9	Tropical	Northern	10.9	SHR
Malaysia	3.1	Tropical	Northern	4.7	NHR
Singapore	1.4	Tropical	Northern	5.8	NHR
Uganda	0.3	Tropical	Northern	10.2	SHR
Ecuador	-0.2	Tropical	Southern	1.5	NHR
Kenya	-1.3	Tropical	Southern	3.3	NHR
Rwanda	-2.0	Tropical	Southern	3.6	NHR
D. R. of the Congo	-4.3	Tropical	Southern	1.8	NHR
Congo	-4.7	Tropical	Southern	6.4	SHR
Tanzania	-6.2	Tropical	Southern	1.2	NHR
Indonesia	-6.2	Tropical	Southern	1.7	NHR
Angola	-8.8	Tropical	Southern	4.0	NHR
Peru	-12.0	Tropical	Southern	8.0	SHR
Zambia	-15.4	Tropical	Southern	9.2	SHR
Bolivia	-16.5	Tropical	Southern	6.5	SHR

**Fiji**	**-18.1**	**Tropical**	**Southern**	10.1	**SHR**
**Madagascar**	**-18.9**	**Tropical**	**Southern**	2.4	**NHR**
**Mauritius**	**-20.2**	**Tropical**	**Southern**	7.5	**SHR**
**New Caledonia**	**-22.3**	**Tropical**	**Southern**	8.5	**SHR**
Paraguay	*-25.3*	*Temperate*	Southern	7.2	**SHR**
South Africa	*-25.7*	*Temperate*	Southern	7.3	**SHR**
Mozambique	*-26.0*	*Temperate*	Southern	3.2	**NHR**
Chile	*-33.5*	*Temperate*	Southern	7.7	**SHR**
Argentina	*-34.6*	*Temperate*	Southern	7.4	**SHR**
Uruguay	*-34.9*	*Temperate*	Southern	7.7	**SHR**
Australia	*-33.9*	*Temperate*	Southern	8.3	**SHR**
New Zealand	*-41.3*	*Temperate*	Southern	8.3	**SHR**

* based on the latitude of the capital
** second digit denotes the time within the month
